# Elevated Cancer Antigen 125 (CA125) Levels in an Indian Male: A Diagnostic and Therapeutic Marker in Extrapulmonary Tuberculosis

**DOI:** 10.7759/cureus.105575

**Published:** 2026-03-21

**Authors:** Tejas V M, Akash B R, Veena R M

**Affiliations:** 1 Internal Medicine, BGS Global Institute of Medical Sciences, Bangalore, IND; 2 Pharmacology, BGS Global Institute of Medical Sciences, Bangalore, IND

**Keywords:** antitubercular therapy, ascites, ca125, case report, diagnostic marker, extrapulmonary tuberculosis, prognostic marker, treatment, tuberculosis, tuberculous peritonitis

## Abstract

A patient with ascites and evening chills presenting to OPD was suspected of having tuberculosis (TB) peritonitis after ruling out possible diagnoses. Abdominal paracentesis for molecular studies, including PCR and CBNAAT (cartridge-based nucleic acid amplification test), was negative. Based on previous evidence and investigation findings from our case, we report the value and application of cancer antigen 125 (CA125) in the diagnosis and follow-up of this case of extrapulmonary TB. This case highlights the potential role of CA125 as a supportive diagnostic and prognostic marker in extrapulmonary TB. The non-invasive and cost-effective nature of CA125 may aid in monitoring anti-tubercular therapy response, an alternative to invasive investigation techniques.

## Introduction

Tuberculosis (TB) remains a major global public health concern, with an estimated 10.7 million incident cases and 1.23 million deaths reported worldwide in 2024, and India accounting for approximately 25% of the global TB burden, making it the highest contributor among high-burden countries according to the WHO Global Tuberculosis Report 2025 [1].

Patients with extrapulmonary TB (EPTB) are more likely to have negative smear results, and many EPTB cases do not involve the lungs directly [2]. Tuberculous peritonitis is one of the common extrapulmonary locations, accounting for around 6% of EPTB cases [3]. This manifestation of TB is one of the most challenging forms of EPTB to diagnose [2].

Carbohydrate antigen 125 (CA125) is a high-molecular-weight glycoprotein expressed by a large proportion of epithelial ovarian cancers. It is detected using the OC125 monoclonal antibody, first described by Moss et al. in 1981 [4]. Since its discovery, CA125 has become a well-established tumor marker for epithelial ovarian cancer and plays an important role in diagnosis, including incorporation into the risk of malignancy index [4]. Elevated CA125 levels have also been observed in patients with peritoneal TB [5].

Under physiological conditions, CA125 is expressed on the cell membrane but does not cross into the bloodstream due to intact cell junctions. Pathological states that disrupt this barrier result in shedding of the antigen into the blood, causing a serological rise in CA125 levels [6].

Based on this evidence, we explored the role of CA125 in a male patient who was sputum- and PCR-negative and did not exhibit granuloma formation. This report describes the evaluation of CA125 in diagnosing tuberculous peritonitis and the observed decline in CA125 levels during antitubercular therapy, marking the end of treatment and resolution of chronic peritoneal irritation caused by *Mycobacterium tuberculosis*.

## Case presentation

**Figure 1:**
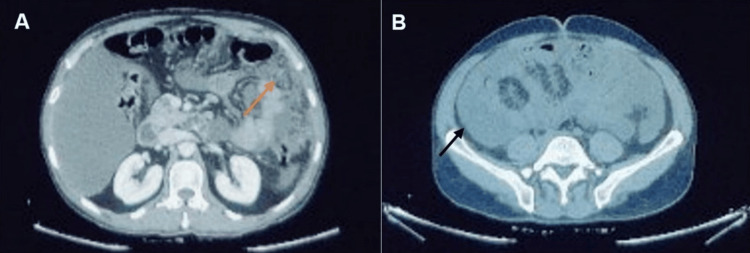
Computed tomography imaging findings obtained at hospital admission Contrast-enhanced computed tomographic axial images showing (A) thickening and nodularity of the peritoneum and omentum (orange arrow) and (B) presence of pelvic ascites (black arrow)

A 45-year-old man presented to our OPD in Bengaluru, Karnataka, on a referral basis regarding his distended abdomen. He was apparently normal three months back and later on developed abdominal distension ever since, which was gradually progressive. He had lost his weight by 10 kg (from 73 kg to 63 kg) in three months with a history of low-grade fever with an evening rise in temperature. His initial consultation was two months ago, after which he received medical attention at multiple healthcare facilities before arriving at our facility. He underwent liver function tests (LFTs), renal function tests (RFTs), and a computed tomography (CT) scan of the abdomen and thorax before his visit here, which revealed enlargement of the para-aortic and mesenteric lymph nodes, along with subcarinal, periesophageal, and cardiophrenic lymph nodes. Thickening and nodularity of the peritoneum and omentum were observed, as shown in Figure 1A. Ascites was also detected, as visualized in Figure 1B. Serological tests for hepatitis B virus (HBV) and hepatitis C virus (HCV) were negative. Liver and RFTs were within normal limits. Hematology reports showed normal alpha-fetoprotein (AFP) levels, as shown in Table 1.

**Table 1:** Tumor marker levels at the time of admission AFP: alpha-fetoprotein; CA125: cancer antigen 125

Investigation	Results	Reference Intervals
AFP	2.0 IU/mL	<7.0 IU/mL
CA125	173.3 IU/mL	<35 IU/mL

Upon admission to our facility, clinical examination revealed abdominal distension with shifting dullness and the presence of bowel sounds. The abdomen was soft and non-tender, and no evidence of organomegaly was noted. There were no signs of any palpable mass. Abdominal paracentesis was performed, and cytological examination of the ascitic fluid reported negative for malignant cells. The ascitic fluid analysis showed elevated levels of adenosine deaminase (ADA), lactate dehydrogenase (LDH), and protein, as shown in Table 2. Increased numbers of lymphocytes and mesothelial cells were also observed (Table 3). Although imaging findings raised the possibility of peritoneal malignancy, negative ascitic fluid cytology and normal tumor markers favored the diagnosis of TB over malignancy.

**Table 2:** Biochemical analysis of ascitic fluid LDH: lactate dehydrogenase

Investigation	Results	Reference Intervals
Ascitic fluid sugar	72 mg/dL	50–150.0 mg/dL
Ascitic fluid chloride	101 mEq/L	105–140 mEq/L
Adenosine deaminase	62.4 U/L	Normal: <30 U/L
		Suspect: 30 U/L to 40 U/L
		Strong suspect: >40 U/L to 60 U/L
		Positive: >60 U/L
Ascitic fluid LDH	335	Ascitic fluid LDH/serum LDH: >0.6 indicates exudate
Ascitic fluid protein	5.7 g/dL	1–2.0 g/dL

**Table 3:** Cytological analysis of ascitic fluid

Investigation	Sub-parameters	Results	Reference Intervals
Macroscopy	Volume	25 mL	Variable; depends on the amount aspirated
	Color	Pale yellow	Clear to pale yellow
	Appearance	Clear	Clear
Cell count	-	500 cells/m^3^	<500 cells/m^3^
Cell type	Lymphocytes	85%	Normally <250 cells/m^3^
	Neutrophils	5%	Predominantly mononuclear cells; lymphocyte predominance seen in TB/malignancy
	Mesothelial cells	10%	Present normally
Microscopy	-	Smears studied are cellular, showing predominantly lymphocytes arranged in clusters and singles along with a few mesothelial cells and neutrophils in a proteinaceous and hemorrhagic background. No evidence of atypia/malignancy was seen in the smear studied.	Cytology is descriptive; no standardized reference range
Impression	-	Ascitic fluid—negative for malignancy	Normal: No malignant cells

Although ascitic fluid and sputum AFB (acid-fast bacillus) cultures were negative, the diagnosis of tuberculous peritonitis was strongly suspected based on the clinical presentation, epidemiological background, imaging findings, ascitic fluid analysis, and elevated ADA levels. Therefore, empirical anti-tubercular therapy (ATT) was initiated, and the patient was discharged the next day with advice to follow up.

The patient returned to our center for follow-up to monitor tolerability and potential hepatotoxicity associated with ATT. LFTs revealed an elevated globulin level of 3.6 g/dL and an albumin/globulin (A/G) ratio of 1. Hematological studies showed mild microcytic hypochromic anemia with relative neutrophilia. The patient was advised to continue ATT and to return for a follow-up visit after two months.

**Figure 2:**
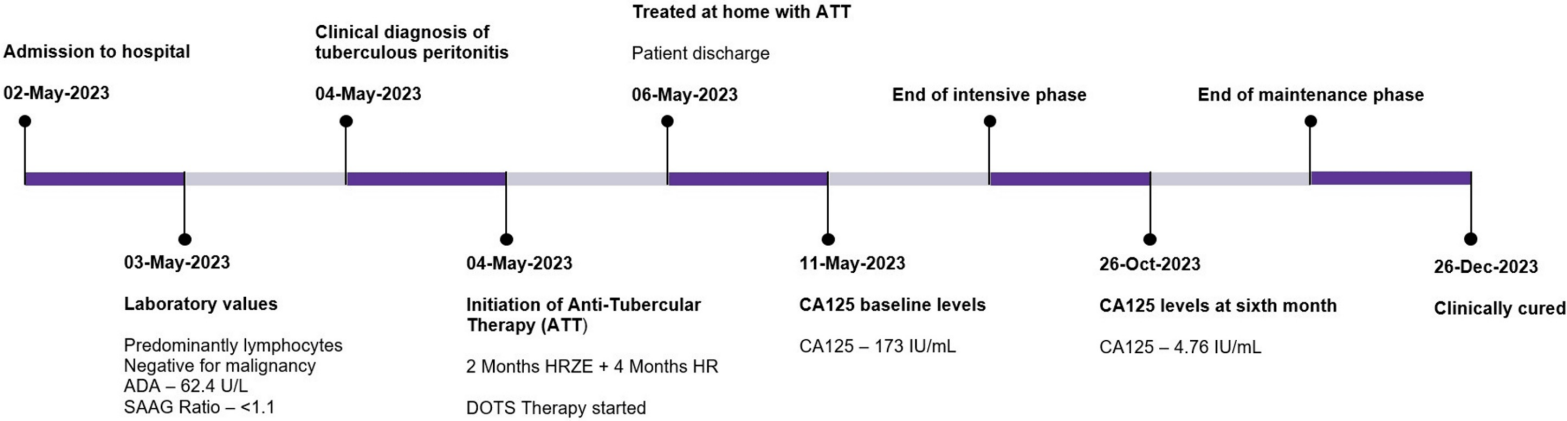
Patient treatment timeline HRZE: isoniazid, rifampin, pyrazinamide, and ethambutol; DOTS: directly observed therapy, short-course; ADA: adenosine deaminase; SAAG: serum–ascitic albumin gradient

The patient was re-assessed after 25 weeks of ATT to evaluate treatment adherence. LFTs revealed mildly elevated serum protein and albumin levels. The patient was advised to continue treatment for an additional two months and to return for a follow-up evaluation. The treatment timeline is visualized in Figure 2.

Results

Before initiating ATT, the CA125 level was elevated fivefold (173.3 IU/mL). By the end of the ATT, CA125 levels had decreased to 4.76 IU/mL (reference range: <35 IU/mL), as shown in Table 4. The patient improved clinically.

**Table 4:** Measurements of CA125 levels throughout the study CA125: cancer antigen 125

Marker	Baseline levels	Levels at the sixth-month follow-up
CA125	173.3 IU/mL	4.76 IU/mL

## Discussion

In this case, we were able to analyze variations in CA125 levels, as stated in the background, as objectives. CA125 is expressed in tissues derived from coelomic epithelia, which include pleura, pericardium, peritoneum, stomach, colon, kidney, ovary, and fallopian tube [7].

Under physiologic conditions, CA125 is expressed on the cell membrane, but the junctional complexes of the cells prevent it from crossing over to the bloodstream. This membrane integrity is disrupted under pathological conditions, leading to the release of this antigen into the blood and, consequently, elevating its serological levels [6].

In challenging cases where the presence of peritoneal TB with obvious ascites may have little or no correlation with pulmonary TB and would demonstrate a sputum-negative condition, CA125 can rule out underlying malignancies and arrive at a diagnosis of peritoneal TB.

Ronay et al. [7] determined that CA125 was immunohistochemically localized and sharply demarcated around tuberculous granuloma in two patients with peritoneal TB. A similar study concluded that a possible explanation for this finding was the inflammatory mesothelial cell proliferation [8].

In our case, no granuloma formation occurred, and sputum cultures, along with molecular tests including CBNAAT and PCR of the ascitic fluid sample for TB bacilli, were negative for TB (Table 5). In this case, ascitic fluid cytology, ADA levels, and SAAG (serum-ascitic albumin gradient) ratio were used, along with CA125, to diagnose peritoneal TB. The patient was adequately followed up, and CA125 levels were measured at each visit. Clinical recovery was evident over time and correlated with a progressive decline in the marker levels, indicating cessation of chronic peritoneal irritation caused by *Mycobacterium tuberculosis*.

**Table 5:** Molecular studies at the time of admission TB-PCR: tuberculosis polymerase chain reaction; CBNAAT: cartridge-based nucleic acid amplification test

Investigation	Results
TB-PCR	Negative
CBNAAT	Negative

Malignancy was considered a possible diagnosis in this patient. Imaging modalities suggested the possibility of either peritoneal malignancy or TB. However, tumor markers such as AFP were within normal limits, and ascitic fluid cytology did not demonstrate malignant cells, making malignancy less likely in this scenario. Advanced investigations such as positron emission tomography-computed tomography (PET-CT) were not performed due to financial constraints. Invasive diagnostic procedures, including diagnostic laparoscopy with lymph node biopsy, were also not pursued due to their invasive nature and the associated financial burden for the patient. These investigations are typically considered later in the diagnostic workup after initial assessments such as ultrasonography, contrast-enhanced computed tomography (CECT), ascitic fluid analysis, and tumor marker evaluation [9]. Furthermore, the patient demonstrated significant symptomatic and clinical improvement following initiation of ATT, which further supported the diagnosis of tuberculous peritonitis rather than malignancy.

CA125 demonstrates high sensitivity and specificity in the context of peritoneal TB, 97.5% and 100%, respectively [7]. Similarly, ADA has also been shown to possess high diagnostic sensitivity and specificity in peritoneal TB [10]. CA125 may be utilized as an adjunctive marker alongside ADA or even considered a cost-effective mainstay marker alternative to invasive procedures for both diagnosis and prognosis in cases of peritoneal TB. Its role is particularly important in monitoring treatment response and assessing disease progression during ATT. This case further supports the application of CA125 in monitoring treatment response and patient recovery.

Limitations and scope

The limitation of the study was that the advanced diagnostic investigations, such as PET-CT and histopathological confirmation through diagnostic laparoscopy with lymph node biopsy, were not performed due to financial constraints and the invasive nature of the procedures. A definitive histopathological tissue-based diagnosis could not be obtained. This limited us to diagnosing the patient solely on his history, clinical examination, laboratory findings, imaging, and a favorable response to ATT.

Further studies with larger sample sizes and frequent, timely CA125 measurements, along with correlations with standardized tests, can lay a benchmark for its use as a cheaper yet significant prognostic marker.

## Conclusions

On the grounds that ovarian carcinomas are of nil possibility of diagnosis in males, ascitic fluid cytology, ADA levels, and the SAAG ratio ruled out underlying malignancy, establishing a diagnosis of peritoneal TB. CA125 has a coelomic epithelial origin and need not be present only in females but also in males. Based on follow-up of this patient, he had significantly recovered clinically, which can be directly correlated to the drop in CA125 levels, indicating cessation of chronic irritation of the peritoneal tissues caused by *Mycobacterium tuberculosis*.
